# Nitrogen assimilation and photorespiration become more efficient under chloride nutrition as a beneficial macronutrient

**DOI:** 10.3389/fpls.2022.1058774

**Published:** 2023-01-10

**Authors:** Procopio Peinado-Torrubia, Rosario Álvarez, Marta Lucas, Juan D. Franco-Navarro, Francisco J. Durán-Gutiérrez, José M. Colmenero-Flores, Miguel A. Rosales

**Affiliations:** ^1^ Plant Ion and Water Regulation Group, Instituto de Recursos Naturales y Agrobiología de Sevilla (IRNAS, CSIC), Seville, Spain; ^2^ Departamento de Biología Vegetal y Ecología, Facultad de Biología Universidad de Sevilla, Sevilla, Spain; ^3^ Laboratory of Plant Molecular Ecophysiology, Instituto de Recursos Naturales y Agrobiología de Sevilla (IRNAS, CSIC), Seville, Spain

**Keywords:** amino acids, nitrate, metabolism, nitrogen use efficiency, tobacco, NUE

## Abstract

Chloride (Cl^−^) and nitrate ( ${\text{NO}}_{3}^{-}$ ) are closely related anions involved in plant growth. Their similar physical and chemical properties make them to interact in cellular processes like electrical balance and osmoregulation. Since both anions share transport mechanisms, Cl^−^ has been considered to antagonize ${\text{NO}}_{3}^{-}$ uptake and accumulation in plants. However, we have recently demonstrated that Cl^−^ provided at beneficial macronutrient levels improves nitrogen (N) use efficiency (NUE). Biochemical mechanisms by which beneficial Cl^−^ nutrition improves NUE in plants are poorly understood. First, we determined that Cl^−^ nutrition at beneficial macronutrient levels did not impair the ${\text{NO}}_{3}^{-}$ uptake efficiency, maintaining similar ${\text{NO}}_{3}^{-}$ content in the root and in the xylem sap. Second, leaf ${\text{NO}}_{3}^{-}$ content was significantly reduced by the treatment of 6 mM Cl^−^ in parallel with an increase in ${\text{NO}}_{3}^{-}$ utilization and NUE. To verify whether Cl^−^ nutrition reduces leaf ${\text{NO}}_{3}^{-}$ accumulation by inducing its assimilation, we analysed the content of N forms and the activity of different enzymes and genes involved in N metabolism. Chloride supply increased transcript accumulation and activity of most enzymes involved in ${\text{NO}}_{3}^{-}$ assimilation into amino acids, along with a greater accumulation of organic N (mostly proteins). A reduced glycine/serine ratio and a greater ammonium accumulation pointed to a higher activity of the photorespiration pathway in leaves of Cl^−^-treated plants. Chloride, in turn, promoted higher transcript levels of genes encoding enzymes of the photorespiration pathway. Accordingly, microscopy observations suggested strong interactions between different cellular organelles involved in photorespiration. Therefore, in this work we demonstrate for the first time that the greater ${\text{NO}}_{3}^{-}$ utilization and NUE induced by beneficial Cl^−^ nutrition is mainly due to the stimulation of ${\text{NO}}_{3}^{-}$ assimilation and photorespiration, possibly favouring the production of ammonia, reductants and intermediates that optimize C-N re-utilization and plant growth. This work demonstrates new Cl^−^ functions and remarks on its relevance as a potential tool to manipulate NUE in plants.

## Introduction

Nitrogen (N) is an essential macronutrient for plant growth and development (Maathuis, 2009), required for the biosynthesis of a wide range of crucial molecules such as amino acids, chlorophylls, lipids, signal molecules, or secondary metabolites (Britikov et al., 1970; Kusano et al., 2011; Guan, 2017). In agriculture, N availability represents a major bottleneck for crop yield, leading to abusive use of N-rich fertilizers with dramatic consequences for the environment, food quality and human health (Smolders et al., 2010; Musacchio et al., 2020). Since plants only use 30-40% of N applied to soils, a greater N use efficiency (NUE) is required to improve the sustainability, yield and quality of crops (Bijay and Craswell, 2021). NUE is an agronomic parameter defined as the plant biomass produced relative to the N available in the soil (Moll et al., 1982; Xu et al., 2012). NUE can depend on the plant ability to either take up N from the soil (i.e., N-uptake efficiency; NU_P_E) or to convert the acquired N to biomass or grain yield (N-utilization efficiency; NU_T_E). These parameters are considered important traits in agriculture, since crops with higher NUE promote greater yields under limited N in soil, or require lower N fertilization to reach similar yield. Therefore, new strategies to enhance NUE in plants are required to reduce the abusive use of N fertilizers or to improve plant growth when low N is available, with substantial benefits for farmers and to the environment (Baligar et al., 2001; Kant et al., 2011; Han et al., 2016).

In aerobic soils, nitrate ( ${\text{NO}}_{3}^{-}$ ) is the main source of N and also a limiting nutrient for adequate plant growth and development (Wang et al., 2012; Krapp et al., 2014; Weber & Burow, 2018). ${\text{NO}}_{3}^{-}$ is taken up by roots through fine-tuned high affinity (HATS) and low affinity (LATS) transport systems (e.g., plasma membrane proteins from the NRT2 and NRT1 gene families, respectively (Carillo and Rouphael, 2022). Subsequently, ${\text{NO}}_{3}^{-}$ can be directly assimilated in root tissues or transported through xylem vessels to aerial organs to be further assimilated or stored in the big vacuoles of the photosynthetic tissues (Crawford, 1995). The N assimilation pathway implies several steps: i) the reduction of ${\text{NO}}_{3}^{-}$ to nitrite ( ${\text{NO}}_{2}^{-}$ ) by the ${\text{NO}}_{3}^{-}$ reductase (NR) in the cytosol; ii) the conversion of ${\text{NO}}_{2}^{-}$ to ammonium ( ${\text{NH}}_{4}^{+}$ ) by the ${\text{NO}}_{2}^{-}$ reductase (NiR) in the chloroplast; iii) the incorporation of ${\text{NH}}_{4}^{+}$ into a photosynthetic carbon (C) backbone through the coordinated activity of the glutamine synthetase (GS) and the glutamate synthase (GOGAT) enzymes, also in the chloroplast; and, ultimately, iv) the cytosolic glutamate dehydrogenase (GDH), asparagine synthetase (ASN) and aspartate aminotransferase (AAT) enzymes are involved in the exchange of N molecules for the biosynthesis of different amino acids (Crawford et al., 2000; Sánchez-Rodríguez et al., 2011; Iqbal et al., 2020; Lee et al., 2020).

Both N and C assimilation processes are tightly regulated and synchronized for optimal biosynthesis of macromolecules such as proteins and nucleic acids (Baslam et al., 2021). The assimilation of N and C must be adequately adjusted to adapt plant growth according to environmental conditions. Photorespiration initiates simultaneously with photosynthesis in the chloroplast as a result of the oxygenation of the ribulose-1,5-biphosphate (RuBP) by the RuBP carboxylase/oxygenase (RuBisCO), generating glycerate-3-phosphate (3-PGA) and glycolate-2-phosphate (2-PG). The 2-PG is hydrolyzed to glycolate by the phosphoglycolate phosphatase (PGP), transported to the peroxisome to be oxidized to glyoxylate by the glyoxylate oxidase (GO) and transaminated to glycine (Gly) by glutamate:glyoxylate aminotransferase (GGAT). In the mitochondrion, two molecules of Gly convert into serine (Ser) by the combined activity of the enzymes glycine decarboxylase (GDC) and serine hydroxymethyltransferase (SHMT), releasing CO_2_, ${\text{NH}}_{4}^{+}$ , and reducing power (NADH). Ser is transported to the peroxisome and converted in hidroxypyruvate and glycerate by the action of serine:glyoxilate aminotransferase (SGAT) and hydroxypyruvate reductase (HPR), respectively. In the chloroplast, glycerate results in glycerate-3-phosphate by the glycerate kinase (GK), which is finally converted to RuBP in the Calvin cycle (Wingler et al., 2000). Thus, both C and N metabolisms remain connected in leaves by the formation of ${\text{NH}}_{4}^{+}$ from mitochondria and α-ketoglutarate (αKG) from peroxisomes, which are transported into chloroplasts to be re-assimilated *via* the GS/GOGAT cycle. Recently, photorespiration has been proposed as a compensatory mechanism by which plants that depend on ${\text{NO}}_{3}^{-}$ as the sole N source use 25% of their photo-assimilates to avoid protein depletion (Bloom, 2015). As a by-product, the photorespiratory pathway generates ${\text{NH}}_{4}^{+}$ up to 20 times more than that resulting from ${\text{NO}}_{3}^{-}$ reduction (Guo et al., 2007; Shi and Bloom, 2021). Therefore, photorespiration arises as a relevant physiological process that might optimize NUE by specifically providing intermediates and reducing molecules to concomitantly assure energy dissipation and photoprotection and enhancing N assimilation (Wingler et al., 2000). ${\text{NO}}_{3}^{-}$ and chloride (Cl^−^) are the most abundant inorganic anions in plants, but contrary to ${\text{NO}}_{3}^{-}$ , Cl^−^ is not metabolized. Both anions share very similar physical properties in solution, playing important roles in charge balance and cell osmoregulation, showing strong dynamic interactions in land plants (Wege et al., 2017).

Cl^−^ is an essential micronutrient since it is needed in small quantities for healthy growth of plants (<50-100 μM in the nutrient medium; Xu et al., 2000; White and Broadley, 2001). Cl^−^ is involved in the stabilization of the water splitting system of photosystem-II and the regulation of enzyme activities. Important Cl^−^ functions are also related to osmoregulation, electrical charge balance of cations, stabilization of the electric potential of cell membranes and regulation of pH gradients. However, plants take up Cl^−^ very actively, accumulating it in much higher concentrations (50-500 times) than those required as a micronutrient (Brumós et al., 2010; Franco-Navarro et al., 2016). As an anion, this implies a high cost of metabolic energy (Brumós et al., 2010). When supplied to levels in excess to satisfy micronutrient requirements but insufficient to cause toxicity (i.e., 1-5 mM Cl^−^), Cl^−^ is accumulated at typical macronutrient concentrations, promoting plant growth by specifically improving the efficiency in the use of water, N and C, the main components of plant nutrition (Franco-Navarro et al., 2016; Franco-Navarro et al., 2019; Rosales et al., 2020), giving rise to the recent definition of Cl^−^ as a beneficial macronutrient (Colmenero-Flores et al., 2019).

Considering the reported close interactions between ${\text{NO}}_{3}^{-}$ and Cl^−^, we recently uncovered positive correlations between leaf Cl^−^ content vs. NUE and plant growth in different species of agronomic interest (Rosales et al., 2020). These results point out that Cl^−^ accumulation at macronutrient levels somehow reduces ${\text{NO}}_{3}^{-}$ sequestration in leaves and, therefore, makes it more available for assimilation. However, the role of beneficial Cl^−^ nutrition on the ${\text{NO}}_{3}^{-}$ allocation and assimilation processes remain unknown. Therefore, in this work we further assessed the unknown effect of beneficial Cl^−^ nutrition on ${\text{NO}}_{3}^{-}$ allocation and N metabolism. Our results demonstrate for the first time that Cl^−^ improves NUE by promoting ${\text{NO}}_{3}^{-}$ assimilation through the simultaneous stimulation of both ${\text{NO}}_{3}^{-}$ reduction (via NR activity) and ${\text{NH}}_{4}^{+}$ production from the photorespiration pathway, without altering the ${\text{NO}}_{3}^{-}$ uptake and transport in tobacco plants.

## Material and methods

### Plant material and nutritional treatments

Seeds of tobacco (*Nicotiana tabacum* L. var. Havana) were sown in flat trays (cell size, 4 cm × 4 cm × 10 cm) and grown under experimental greenhouse conditions with controlled temperature between 25 ± 3/18 ± 1 °C (day/night), a relative humidity of 60 ± 10% (EL 1-USB Data logger Lascar Electronics Inc., Erie, PA, USA), 16/8 h photoperiod with a photosynthetic proton flux density (average photosynthetically active radiation; PAR) of 300–350 μmol m^–2^ s^–1^ (quantum sensor, LI-6400; Li-COR, Lincoln, NE, USA), and a luminous emittance of 9,000–10,000 lx (Digital Lux Meter, LX1010B; Carson Electronics, Valemount, Canada). At 15 days after sowing (DAS), seedlings were transferred to 7.5 L pots (20 cm x 17 cm x 25 cm) with a soil substrate containing a mixture of washed vermiculite:perlite (7:3). During the experiments, plants were watered with a basal nutrient solution containing: 4 mM KNO_3_, 1 mM Mg(NO_3_)_2_, 1 mM Ca(NO_3_)_2_, 0.1 mM FeNa-EDTA, 50 μM KCl, 0.1 mM H_3_BO_3_, 0.1 mM MnSO_4_, 29 μM ZnSO_4_, 0.11 μM CoCl_2_ 0.1 μM CuSO_4_, 1 μM Na_2_MoO_4_, and 5 μM KI. In addition, three nutritional treatments were applied to evaluate the effect of Cl^−^ nutrition: 0 mM Cl^−^ (0CL; basal solution containing 0.075 mM Cl^−^), 2 mM Cl^−^ (2CL; 1 mM KCl + 0.25 mM MgCl_2_ + 0.25 mM CaCl_2_), and 6 mM Cl^−^ (6CL; 3 mM KCl + 0.75 mM MgCl_2_ + 0.75 mM CaCl_2_). To counterbalance all cations supplemented with the three CL treatments and to fulfil plant nutritional requirements, phosphate and sulphate salts were added to 0CL, 2CL and 6CL treatments as follows: 4.32, 3.24 and 1.08 mM KH_2_PO_4_; 0.48, 0.36 and 0.12 K_2_HPO_4_; 1.6, 1.2 and 0.4 mM MgSO_4_; and 1.6, 1.2, and 0.4 mM CaSO_4_; respectively. Considering that 50 µM Cl^−^ was reported to ensure Cl^−^ micronutrient requirements in different plant species (Johnson et al., 1957), 75 µM Cl^−^ was present in the basal nutrient solution (added as 50 μM KCl and 0.11 μM CoCl_2_, including water traces) to fulfil micronutrient Cl^−^ functions in low Cl^−^ treatments (Franco-Navarro et al., 2016; Franco-Navarro et al., 2019). All nutrient solutions were adjusted to pH 5.7 with KOH.

Pots were watered every two days, allowing adequate drainage to keep the soil close to field capacity (3.5 mL g^−1^ substrate) and to avoid both water deficit and flooding events during the course of the experiments. When the flowering stage started (60 DAS), half of the plants used in the assay were randomly harvested and weighted before noon (between 10:00-12:00). This material was further divided into two parts: one part was frozen with liquid nitrogen and conserved at -80°C in an ultra-low temperature freezer for further measurements of enzymatic activities; the other part was dried in a forced-air oven at 75°C for 48-72 hours, grounded to powder in a mortar and preserved dry for further recording of plant organ dry weight (DW) and for determination of different nutrient contents. The other half of the plant samples were used for xylem sap extraction and ion content determination.

Experiments were performed in three independent times, showing in figures results from one representative experiment (*n* = 6-12), which confirmed similar tendencies between the three independent experiments.

### Xylem sap quantification

Xylem sap was collected from tobacco plants of 60 DAS grown in the 0CL (containing 75 uM of Cl^–^), 2CL and 6CL treatments. Pots were transferred to a airtight container, and submerged in the appropriate nutrient solution almost up to substrate level. Each plant was detached using a razor blade, and the root sap exudate was collected 1h later. Shoots were harvested and weighted to obtain fresh weight, and samples were dried in a force-oven at 75°C for 48-72 hours to obtain dry weight.

Xylem sap samples were dilluted in a rate of 1:3. For the determination of anion content, colorimetric assays were conducted using a microplate spectrophotometer reader (Omega SPECTROstar, BMG LABTECH GmbH, Germany).

### Ion content and nitrogen use efficiency parameters

Oven-dried tissues from fully photosynthetic and expanded leaves were ground to powder at room temperature and aqueous extracts were obtained for the determination of Cl^−^, ${\text{NO}}_{3}^{-}$ and ${\text{NH}}_{4}^{+}$ contents, as previously reported in Ruiz et al. (2006) and Franco-Navarro et al. (2016). ${\text{NH}}_{4}^{+}$ was determined from the aqueous extraction by using the colorimetric method described by Krom (1980). Organic N was determined by the Kjeldahl method (Bradstreet, 1954), which the ${\text{NH}}_{4}^{+}$ content was subtracted. Total N content (TNC) represents the sum of organic N and the N that forms part of both ${\text{NH}}_{4}^{+}$ and ${\text{NO}}_{3}^{-}$ molecules, expressed as mg g^−1^ DW. Total N accumulation (TNA) was calculated as the result of TNC multiplied by total DW as described in Rubio-Wilhelmi et al. (2011), and results were expressed as mg of N. NUE is commonly defined as vegetative yield per unit of N available in the soil (g DW g^−1^ N; Moll et al., 1982), and can be subdivided into two types: (i) N utilization efficiency (NU_T_E) calculated as total DW divided by TNC (g^2^ DW mg^−1^ N; Siddiqi and Glass, 1981) and (ii) N uptake efficiency (NU_P_E) calculated as TNA divided by root DW (g N g^−1^ root DW; Elliott and Läuchli, 1985).

### Enzymatic assays

Frozen leaf tissues were ground in a chilled mortar with 1 mL of extract buffer (pH 7.5), which contained 50 mM KH_2_PO_4_, 2 mM ethylenediaminetetraacetic acid (EDTA), 2 mM dithiothreitol (DTT) and 1% (w/v) polyvinylpolypyrrolidone (PVPP). Homogenate was centrifuged for 20 min at 30,000 *g*. The upper fraction was used to quantify nitrate reductase (NR; EC 1.7.99.4), nitrite reductase (NiR; EC 1.7.2.1), glutamate synthase (GOGAT; EC 1.4.7.1), and glutamate dehydrogenase (GDH; EC 1.4.1.2) by spectrophotometry, following methods described by Sánchez-Rodríguez et al. (2011) with slight modifications. For glutamine synthetase (GS; EC 6.3.1.2) and aspartate aminotransferase (AAT; EC 2.6.1.1) activities, frozen leaf tissue were ground in 1 mL of extract buffer maleic acid KOH (pH 6.8), which included 100 mM sucrose, 2 mM DTT, and 20% (v/v) ethylene glycol. Homogenate was centrifuged at 30,000 *g* for 20 min. Supernatant was used for colorimetric and kinetic assays (GS and AAT, respectively), based on methods described by Wallsgrove et al. (1979); Kaiser and Lewis (1984) and González et al. (1995) with slight modifications. All these activities are shown in **Figure S1**.

Protein assay was done according to the colorimetric method Bradford (1976) using bovine serum albumin (BSA) as standard.

### Quantification of soluble amino acids

Lyophilized leaf tissues (2 mg) were powdered and homogenized in 1 mL ethanol:water (3:7 v/v). The mixture was stirred for 30 minutes at room temperature and centrifuged at 8,850 *g* for 10 minutes. Samples were derivatized by reaction with diethyl ethoxymethylenemalonate and analyzed by reverse-phase high-performance liquid chromatography as described by Megías et al. (2015).

### Total RNA isolation and analysis

Total RNA was extracted from ground leaf samples using TRIsure reagent procedure (BIOLINE, London, UK). One µg of total RNA was used as a template for first strand cDNA synthesis in a 10 μl reaction using the iScript cDNA synthesis kit (BIO-RAD, Hercules, CA, USA). Diluted cDNA was used as a template for gene expression level quantification by quantitative real-time reverse transcription (Q-RT)-PCR. The iTaq Universal SYBR Green Supermix (BIO-RAD) was used for Q-PCR analyses, using a CFX Connect Real-Time Detection System (BIO-RAD Laboratories) and following the manufacturer’s instructions. PCR was carried out with previously designed gene-specific primers (**Tables S1**, **S2**). Data were analysed using the Bio-Rad CFX Manager software. Six biological replicates and two technical repeats were used per treatment in every run. All mRNA levels were calculated from threshold cycle values and normalized with respect to the transcript level of the housekeeping genes *NtL25 (Nitab4.5_0002995g0050.1)* and *NtGAPGH (Nitab4.5_0005327g0090.1)*. Reactions were performed on two independent RNA batches and results were comparable in the different assays. Results were also given as “Fold Change” when the transcript abundance of the treatment was divided by the control value.

### Electron microscopy

For transmission electron microscopy (TEM), leaves sections (60 DAS) around 1–2 mm^2^ were fixed for 24h at room temperature in 4% (v/v) glutaraldehyde in 0.1 M sodium cacodylate buffer (pH 7.4), and washed in the same buffer. Subsequently, leaf sections were post fixed for 2 h at 4°C in 1% (w/v) osmium tetroxide in 0.1 M sodium cacodylate buffer (pH 7.4) and washed in the same buffer. After fixation, fragments were dehydrated through graded alcohols and embedded in Epon resin (Epoxy Embedding Medium, Sigma). Sections were cut with an ultramicrotome (Reichert-Jung Ultracut) with a diamond knife and mounted on nickel grids. The grids were contrasted with 2% aqueous uranyl acetate and observed with a Zeiss Libra 120 Electron Microscope at 80 kV.

### Statistical analyses

Statistical analysis was performed using STATGRAPHICS Centurion XVI software (StatPoint Technologies, Warrenton, VA, United States). Results were considered non-significant (ns) when *P* > 0.05, and levels of significance were indicated as followed: *P ≤ 0.05; **P ≤ 0.01; ***P ≤ 0.001. The Shapiro–Wilk (*W*) test was used to verify the normality of the datasets; and the Levene test to determine the homogeneity of variance. Data would be transformed if homogeneity of variance or normality could not be achieved using the functions 1/x, √x, and ln(x). Each variable assayed was analysed using one-way Analysis of variance (ANOVA) or multivariate ANOVA (MANOVA) including treatments as grouping factors. Means were compared by Tukey’s HSD *post hoc* test, with different letters indicating significant differences between groups of samples. Whether homogeneity of variance or normality was not reached after data transformation, non-parametric Kruskal-Wallis ANOVA was followed by *post hoc* Mann-Whitney (U test). Results represent the mean of at least six tobacco plants in each treatment (*n* = 6-12), which were reproducible in three independent experiments.

## Results

### Plant biomass and anion content in different plant tissues

**Figure 1:**
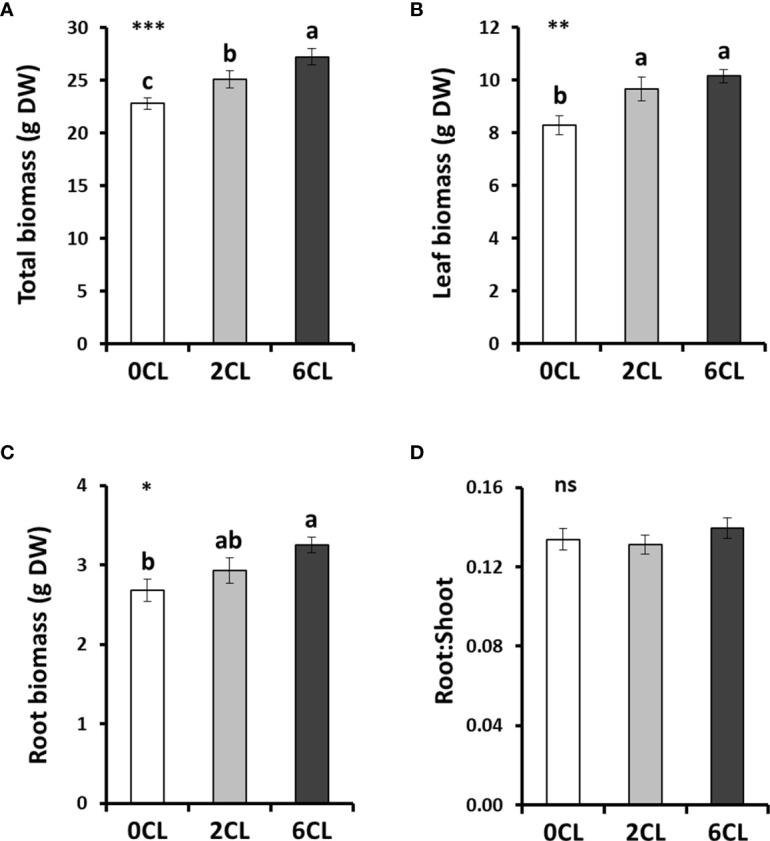
Effect of Cl^−^ nutrition on plant growth. Tobacco plants were treated with 0, 2 and 6 mM of Cl^−^ salts (CL), and supplemented with a mix of sulphate+phosphate salts to maintain a similar cationic balance. **(A)** Total dry weight (DW); **(B)** total leaf DW; **(C)** total root DW; and **(D)** root:shoot ratio are shown. Mean values ± SE, *n*=12. Statistics was calculated through ANOVA, where mean values with different letters are significantly different according to Tukey’s test at *P ≤* 0.05. Levels of significance: *P ≤ 0.05; **P ≤ 0.01; ***P ≤ 0.001; and ‘ns’ *P* > 0.05.

**Figure 2:**
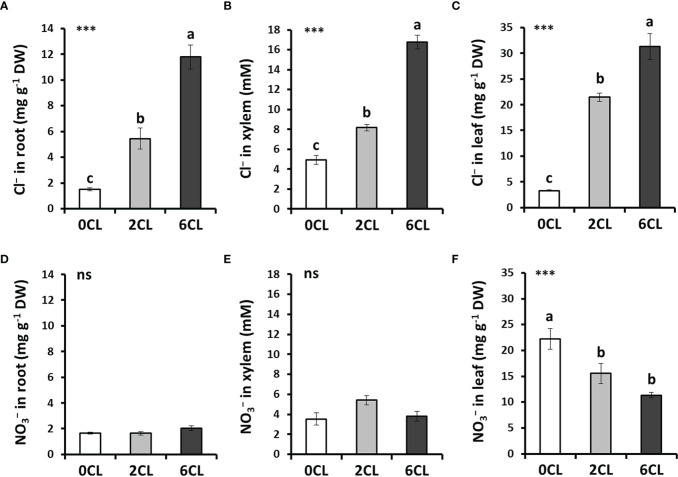
Effect of Cl^−^ nutrition on anion content in different plant tissues. Tobacco plants were treated with 0, 2 and 6 mM of Cl^−^ salts (CL), and supplemented with a mix of sulphate+phosphate salts to maintain a similar cationic balance. Cl^−^ **(A-C)** and ${\text{NO}}_{3}^{-}$ **(D–F)** contents are shown in roots, xylem sap and fully-expanded intermediate leaves, respectively. Mean values ± SE, *n*=6-12. Statistics was calculated through ANOVA, where mean values with different letters are significantly different according to Tukey’s test at *P ≤* 0.05. Levels of significance: ***P ≤ 0.001 and ‘ns’ *P* > 0.05. DW, dry weight.

To study whether Cl^−^ nutrition at beneficial macronutrient levels has a positive impact on N assimilation, we conducted several greenhouse experiments with adult tobacco plants supplemented with 0, 2 and 6 mM Cl^−^ salts (0CL, 2CL, and 6CL, respectively). These nutritional replacements did not induce nutritional deficiencies since no symptoms of wilting, bronzing or chlorosis were observed (**Table S3**; **Figure S4**). Results showed that Cl^−^ treatments promoted plant growth, being 6 mM Cl^−^ (6CL) the treatment that reached the highest values of total, leaf and root biomass (**Figures 1A, C**). However, no significant differences were found in leaf biomass between 2CL and 6CL treatments. The results showed that the increased total biomass was mainly due to higher growth of root and leaf organs (*i.e,* stem and flowers) values (**Figure 1**). This growth stimulation was in line with that previously observed under 1-5 mM Cl^−^ treatments in tobacco plants (Franco-Navarro et al., 2016; Franco-Navarro et al., 2021). No differences in the root:shoot ratio were found between treatments (**Figure 1D**). Consistently, the Cl^−^ content was significantly higher in Cl^−^-treated plants in different plant tissues (i.e., roots, xylem sap and leaves; **Figures 2A–C**), reaching the highest accumulation values in leaves of 6CL plants (31.3 mg g^-1^ DW), which is in agreement with the range of typical macronutrient levels (Franco-Navarro et al., 2016). These results confirmed the beneficial effect of Cl^−^ nutrition at macronutrient levels on the growth of adult tobacco plants. To verify whether Cl^−^ interacts with ${\text{NO}}_{3}^{-}$ nutrition, the ${\text{NO}}_{3}^{-}$ content was also quantified in different plant tissues. Our results showed not significant differences in ${\text{NO}}_{3}^{-}$ accumulation in roots and xylem sap with growing Cl^−^ treatments, and there was no effect in the volume of xylem sap exudates per hour (**Figures 2D, E**, **S4**), suggesting no effects on ${\text{NO}}_{3}^{-}$ uptake and transport. However, significant reductions of ${\text{NO}}_{3}^{-}$ content were found in fully-expanded leaves of 2CL and, particularly, of 6CL plants (about 50%; **Figure 2F**). Interestingly, the strong reduction of leaf ${\text{NO}}_{3}^{-}$ content did not cause any growth impairment in 6CL plants, contrary to what has been traditionally believed regarding the negative effects of Cl^−^ on ${\text{NO}}_{3}^{-}$ nutrition.

### Content of N forms and NUE parameters

**Figure 3:**
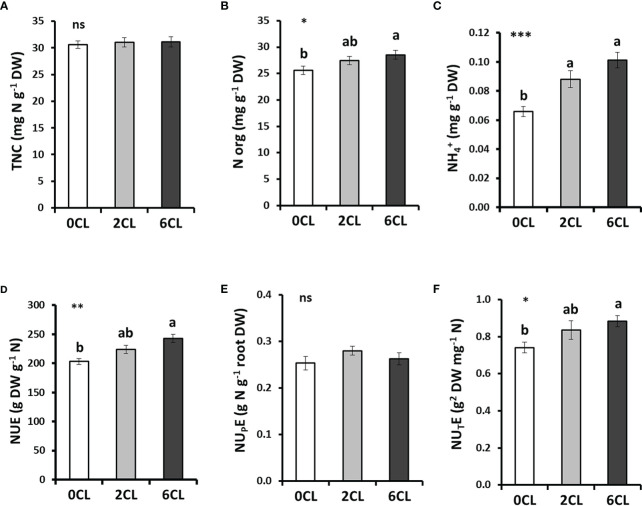
Effect of Cl^−^ nutrition on the content of nitrogen (N) forms and N use efficiency parameters. Tobacco plants were treated with 0, 2 and 6 mM of Cl^−^ salts (CL), and supplemented with a mix of sulphate+phosphate salts to maintain a similar cationic balance. The contents of total N (TNC) **(A)**, organic N (N org) **(B)**, and ammonium ( ${\text{NH}}_{4}^{+}$ ) **(C)** are shown. Nitrogen use efficiency (NUE) **(D)**, N- ${\text{NO}}_{3}^{-}$ uptake efficiency (NU_P_E) **(E)**, and N- ${\text{NO}}_{3}^{-}$ utilization efficiency (NU_T_E) **(F)**. Mean values ± SE, *n*=12. Statistics was calculated through ANOVA, where mean values with different letters are significantly different according to Tukey’s test at *P ≤* 0.05. Levels of significance: *P ≤ 0.05; **P ≤ 0.01; ***P ≤ 0.001; and ‘ns’ *P* > 0.05. DW, dry weight.

To better understand whether the reduction of leaf ${\text{NO}}_{3}^{-}$ content was due to the impact of Cl^−^ on either the uptake or assimilation of ${\text{NO}}_{3}^{-}$ , we first analyzed the content of total and organic N in tobacco leaves (**Figures 3A, B**, **S5C**). Results showed no significant differences in the total N content per biomass unit (TNC) between the three nutritional treatments (0CL, 2CL and 6CL). Interestingly, organic N content significantly increased in 6CL plants, as well as the ${\text{NH}}_{4}^{+}$ content (**Figures 3B, C**, **S5B**). Considering that TNC is mainly composed of organic N (84-92%), N- ${\text{NO}}_{3}^{-}$ (8-16%) and N- ${\text{NH}}_{4}^{+}$ (in a very small proportion, about 0.2%), the strong reduction of ${\text{NO}}_{3}^{-}$ content previously observed in leaves was compensated by the increase of organic N in Cl^−^ -treated plants (**Figure 2F**).

Nutritional treatments (0CL, 2CL and 6CL) were supplied with similar ${\text{NO}}_{3}^{-}$ concentrations in the nutrient solution (8 mM ${\text{NO}}_{3}^{-}$ salts). However, CL plants showed the highest NUE values (**Figure 3D**) despite reducing ${\text{NO}}_{3}^{-}$ accumulation (**Figures 2F**, **S5A**). Therefore, to confirm that the Cl^−^ application promotes a more efficient use of N, two NUE components were determined: i) nitrate uptake efficiency (NU_P_E), indicating how efficiently is N transported into the plant, and ii) nitrate utilization efficiency (NU_T_E), indicating how efficiently the transported N is used by the plant (Moll et al., 1982). Accordingly, no significant differences on NU_P_E values were found in Cl^−^-treated plants (**Figure 3E**), whereas NU_T_E values significantly increased about 20% in 6CL plants (**Figure 3F**).

### Gene expression and activity of enzymes participating in N metabolism

**Figure 4:**
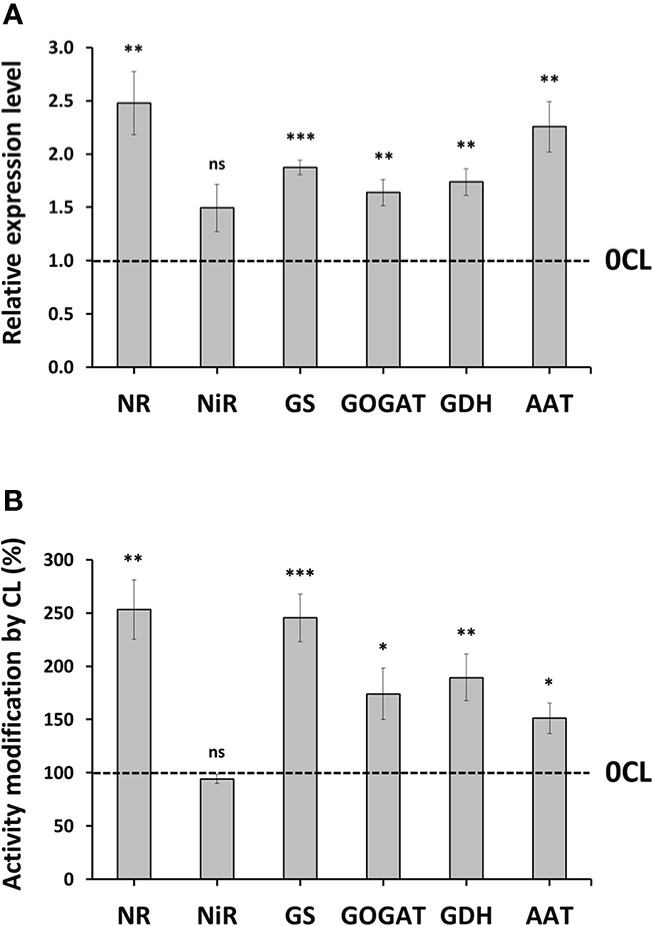
Effect of Cl^−^ nutrition on enzymes involved in N assimilation. Tobacco plants were treated with 0 and 6 mM of Cl^−^ salts (CL), and supplemented with a mix of sulphate+phosphate salts to maintain a similar cationic balance. Transcript abundance **(A)** and activity (%) **(B)** of 6CL plants normalized to 0CL plants showed in different N metabolism-related enzymes: nitrate reductase (NR), nitrite reductase (NiR), glutamine synthetase (GS), glutamate synthase (GOGAT), glutamate dehydrogenase (GDH), and aspartate aminotransferase (AAT). Mean values ± SE, *n*=4-6. Statistics in **(B)** was calculated through ANOVA. Levels of significance: *P ≤ 0.05; **P ≤ 0.01; ***P ≤ 0.001; and ‘ns’ *P* > 0.05. The dotted line represents the normalized activity in 0CL plants.

The strongly decrease in ${\text{NO}}_{3}^{-}$ content in leaves in plants containing higher biomass, organic N content and NUE, suggests that the 6 mM Cl^−^ treatment stimulates the ${\text{NO}}_{3}^{-}$ assimilation into organic molecules. To demonstrate this hypothesis, we quantified the effect of 6 mM Cl^−^ treatment on the transcript abundance and activity of key enzymes involved in ${\text{NO}}_{3}^{-}$ assimilation. In comparison to 0CL plants, the 6 mM Cl^−^ treatment induced higher accumulation of of transcripts encoding for NR, GS, GOGAT, GDH and AAT enzymes, showing lower changes in NiR transcript accumulation (**Figure 4A**). To verify whether the Cl^−^-dependent up-regulation of the genes affected at the post-translational level, the activity of the corresponding enzymes was quantified in *in-vitro* assay using plant extracts. Our results showed significant increases in the activity of all the tested enzymes involved in ${\text{NO}}_{3}^{-}$ assimilation, except for NiR that did not show significant changes (**Figure 4B**). Interestingly, the cytosolic NR, which is the main limiting enzyme of the ${\text{NO}}_{3}^{-}$ assimilation pathway, showed the greatest increase of activity in 6CL plants (253%), followed by the chloroplastic GS and GOGAT enzymes (245% and 174%, respectively). Other cytosolic enzymes involved in the incorporation of N to amino acids and proteins, GDH and AAT, also showed increased activities in 6CL plants (189% and 151%, respectively) (**Figure 4B**).

### Total proteins and amino acids profile

**Figure 5:**
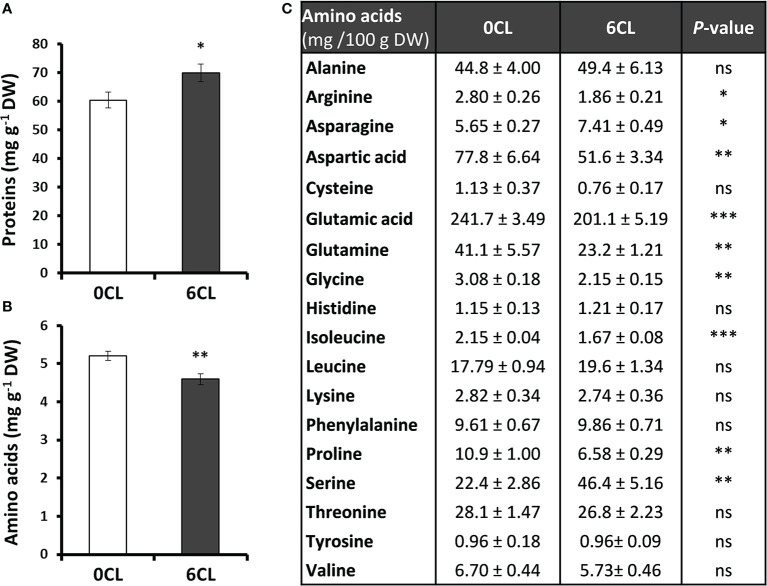
Effect of Cl^−^ nutrition on protein and amino acids content. Tobacco plants were treated with 0 and 6 mM of Cl^−^ salts (CL), and supplemented with a mix of sulphate+phosphate salts to maintain a similar cationic balance. **(A)** Total proteins content; **(B)** Total free amino acids content; **(C)** Amino acids profile. Mean values ± SE, *n*=4-6. Statistics was calculated through ANOVA, where mean values with different letters are significantly different according to Tukey’s test at *P ≤* 0.05. Levels of significance: *P ≤ 0.05; **P ≤ 0.01; ***P ≤ 0.001; and ‘ns’ *P* > 0.05.

Proteins and their elemental units, amino acids, are the major components and sources of organic N in plants. Results showed that the content of total soluble proteins was significantly higher in Cl^−^-treated plants (**Figure 5A**), which is consistent with the higher organic N content shown in the **Figure 3B**. However, the Cl^−^ treatment reduced the content of total soluble amino acids (**Figure 5B**). To better understand the role of Cl^−^ on N metabolism, the profile of the most significant free amino acids in plants was quantified. Thus, the results showed that Cl^−^ promoted a significant accumulation of asparagine and Ser, whereas other amino acids such as arginine, aspartic acid, glutamic acid, glutamine, Gly, isoleucine, and proline were reduced (**Figure 5C**). In addition, no significant differences in the content of alanine, cysteine, histidine, leucine, lysine, phenylalanine, threonine, tyrosine and valine were found (**Figure 5C**).

### Expression patterns of genes encoding enzymes of the photorespiration pathway

**Figure 6:**
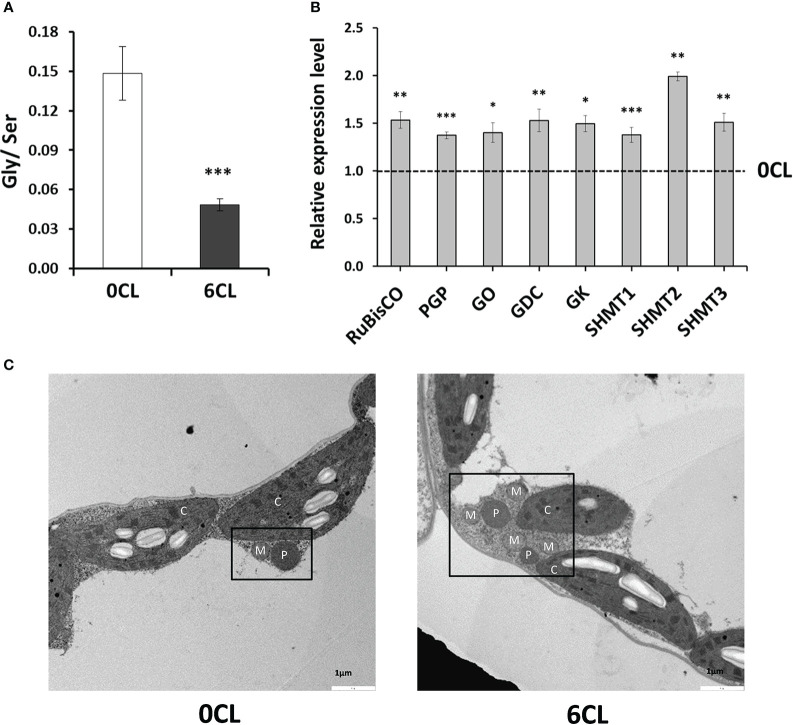
Effect of Cl^−^ nutrition on photorespiration. Tobacco plants were treated with 0 and 6 mM of Cl^−^ salts (CL), and supplemented with a mix of sulphate+phosphate salts to maintain a similar cationic balance. **(A)** Glycine/Serine (Gly/Ser) ratio; **(B)** Relative expression level of photorespiration-related enzymes: ribulose-1,5-biphosphate carboxylase/oxygenase (RuBisCO), phosphoglycolate phosphatase (PGP), glycolate oxydase (GO), glycine decarboxylase (GDC), serine hydroxymethyl transferase (SHMT), glycine kinase (GK). **(C)** Transmission electron micrographs showing the ultrastucture of leaf mesophys cells. Organelles that contribute to photorespiration were labelled as M, mitochondrion; P, peroxisome; C, chloroplast;. Mean values ± SE, *n*=4-6. Statistics was calculated through ANOVA, where mean values with different letters are significantly different according to Tukey’s test at *P ≤* 0.05. Levels of significance: *P ≤ 0.05; **P ≤ 0.01; ***P ≤ 0.001; and ‘ns’ *P* > 0.05.

In this work, the ${\text{NH}}_{4}^{+}$ content increased in leaves of Cl^−^-treated plants (**Figure 3C**). In plants, ${\text{NH}}_{4}^{+}$ is mainly synthetized from two different pathways: i) in chloroplasts through ${\text{NO}}_{3}^{-}$ assimilation and reduction of ${\text{NO}}_{2}^{-}$ *via* NR and NiR; and ii) in mitochondria through the photorespiratory conversion of Gly into Ser, with the latter generating up to 20 times more ${\text{NH}}_{4}^{+}$ (Wingler et al., 2000; Hirel and Lea, 2001). Although we have shown that ${\text{NO}}_{3}^{-}$ assimilation, and consequently ${\text{NH}}_{4}^{+}$ biosynthesis, is stimulated through induction of NR synthesis and activity, the enzymes that participate in ${\text{NH}}_{4}^{+}$ assimilation (GS and GOGAT) were also induced by the Cl^−^-treatment (**Figure 4**). Therefore, we hypothesised whether Cl^−^ exerts a positive effect on the photorespiration pathway. Our results showed a 2-fold increase in the Ser content in CL plants while Gly was significantly reduced (**Figure 5C**), bringing to light a strong reduction in the Gly/Ser ratio (**Figure 6A**). To verify the Cl^−^-dependent effects on the photorespiratory pathway, we determined the abundance of photorespiration-related enzymes transcripts: RuBisCO, PGP, GO, GDC, SHMT, and GK (Busch, 2020). Given the tetraploid nature of *N. tabacum* genome, we conceived three different SHMT candidate genes according to the homology in their *A. thaliana* orthologue gene and the basal gene expression in leaf tissue observed in the Solanaceae Genomics Network (https://solgenomics.net). Interestingly, the expression level of all photorespiration-related genes was up-regulated in CL plants (**Figure 6B**), indicating the Cl^−^-dependent stimulation of the photorespiratory pathway. Furthermore, TEM micrographs showed a higher association of organelles involved in photorespiration (i.e., chloroplasts, peroxisomes and mitochondria) in plants treated with 6CL (**Figure 6C**).

## Discussion

### Beneficial chloride nutrition improves growth andNUEwithoutnegative effects on nitrate allocation

${\text{NO}}_{3}^{-}$ is the main source of N in plants, strongly limiting plant growth and development (Frink et al., 1999; Gojon et al., 2011). The decisive role of N in crop yield has led to excessive use of ${\text{NO}}_{3}^{-}$ in agriculture over decades, which generates serious problems such as water pollution and over-accumulation of ${\text{NO}}_{3}^{-}$ in leaves, becoming harmful to nature and people (Nitrates Directive, 1991; Kant et al., 2011). ${\text{NO}}_{3}^{-}$ and Cl^−^ are the most abundant inorganic anions in plants, but contrary to ${\text{NO}}_{3}^{-}$ , Cl^−^ cannot be metabolized. Both anions share similar physical properties in solution and show strong dynamic interactions in land plants, playing important roles in charge balance and cell osmoregulation (Wege et al., 2017; Colmenero-Flores et al., 2019). For instance, a higher tissue content of Cl^−^ leads to a lower ${\text{NO}}_{3}^{-}$ content and *vice versa* (Xu et al., 2000). This effect has been interpreted as an antagonistic interaction that reduces the plants’ ability to take up ${\text{NO}}_{3}^{-}$ from the soil, given that Cl^−^ and ${\text{NO}}_{3}^{-}$ molecules share transmembrane transport mechanisms with often ambiguous selectivity (Colmenero-Flores et al., 2019). Then, it has traditionally been believed that Cl^−^ alters N nutrition by restricting ${\text{NO}}_{3}^{-}$ transport and accumulation at different levels, dealing to a reduction of NUE in plants (Teakle and Tyerman, 2010; Li et al., 2017; Carillo and Rouphael, 2022). For this reason, and because of the excessive Cl^−^ accumulation in sensitive crops under salinity conditions, Cl^−^ has traditionally been considered detrimental for agriculture. However, we recently defined Cl^−^ as a beneficial macronutrient in higher plants due to its role improving plant growth, water relations, CO_2_ assimilation, water use efficiency and drought resistance, when accumulated in leaves at macronutrient levels (Franco-Navarro et al., 2016; Franco-Navarro et al., 2019 and Colmenero-Flores et al., 2019; Franco-Navarro et al., 2021). Therefore, taking all these beneficial effects into account, we considered unlikely a negative effect of Cl^−^ on ${\text{NO}}_{3}^{-}$ nutrition. Indeed, we recently discovered that Cl^−^ promotes plant growth by improving ${\text{NO}}_{3}^{-}$ utilization and NUE in several species of agronomic interest (Rosales et al., 2020). Consistently, in the present study we observed an increase of total, leaf and root biomass (**Figures 1A–C**) when Cl^−^ accumulates at macronutrient levels in different plant organs (**Figures 2A–C**). Although the Cl^−^ content was markedly lower in roots, xylem and leaves of 0CL plants compared to that of the 6CL treatment (**Figures 2A–C**), it exceeded by 15 times in leaf tissue the critical threshold of deficiency reported in higher plants (about 0.2 mg Cl^−^ g^-1^ shoot DW; Flowers, 1988; Xu et al., 2000; Colmenero-Flores et al., 2019).

Plants utilize metabolic energy to take up nutrients from the soil. To do so, plant cells include semi-permeable plasmatic membranes with transmembrane protein channels for the intake of nutrients. Particularly, for anionic nutrients such as ${\text{NO}}_{3}^{-}$ or Cl^-^, families of H^+^ cotransporters concentrate these nutrients in the root symplast and, subsequently, in the xylem vesicles (Cakmak et al., 2022). Therefore, we ruled out the possibility of Cl^−^ deficiency in 0CL plants since, in addition, no differences between treatments were observed in the root:shoot plant ratios (**Figure 1D**) and no apparent stress symptoms were manifested.

Several studies have reported the antagonism between Cl^−^ and ${\text{NO}}_{3}^{-}$ homeostasis under both high (Buwalda and Smith, 1991; Cerezo et al., 1997; Xu et al., 2000) and mild Cl^−^ salinity (also known as eustress; Colla et al., 2018; Carillo and Rouphael, 2022). It was not clear however whether the Cl^−^-dependent decrease in leaf ${\text{NO}}_{3}^{-}$ content was a consequence of the antagonism exerted by Cl^−^ on ${\text{NO}}_{3}^{-}$ uptake and transport, or an effect on ${\text{NO}}_{3}^{-}$ allocation and assimilation. The long-term accumulation of Cl^−^ in tobacco leaves when supplied at macronutrient levels (far below salinity levels: 2 and 6 mM Cl^−^) did not cause significant reductions in ${\text{NO}}_{3}^{-}$ content in both roots and xylem sap, while the leaf ${\text{NO}}_{3}^{-}$ content was strongly reduced (**Figures 2D–F**). When ${\text{NO}}_{3}^{-}$ is available in the soil, plants normally prioritize the uptake of ${\text{NO}}_{3}^{-}$ over Cl^−^, maintaining active transport mechanisms that are more selective for ${\text{NO}}_{3}^{-}$ than for Cl^−^ (Wege et al., 2017; Wen et al., 2017). Therefore, considering the plant preference for ${\text{NO}}_{3}^{-}$ uptake, our results are consistent with the fact that in the presence of 8 mM ${\text{NO}}_{3}^{-}$ , the supply of 2 and 6 mM Cl^−^ in the nutrient solution does not antagonizes for the selective mechanisms of ${\text{NO}}_{3}^{-}$ uptake and transport, although more research is needed in this regard.

In agriculture, N fertilization represents a major bottleneck for crop yield due to its well-known crucial role in plant growth and metabolic processes. The high energy cost for the synthesis of N fertilizers, as well as its intrinsic mobility in the complex atmosphere-plant-soil system, have highlighted the environmental drawbacks of the unsustainable N use in agriculture (Rothstein, 2007; Keeney and Hatfield, 2008; Garnett et al., 2009; Bijay and Craswell, 2021). Since only 30-40% of the N applied to soils is used by plants, a higher NUE could improve crop yield and quality, while reducing economic costs and decreasing environmental degradation (Baligar et al., 2001). Therefore, NUE parameters (specifically NUE and NU_T_E) are considered important crop traits and a potential tool to reduce the abusive use of N fertilizers or to improve plant growth when low N is available (Baligar et al., 2001; Kant et al., 2011; Han et al., 2016). In this work, our results in tobacco plants confirmed the stimulation of NUE and NU_T_E through Cl^−^ nutrition (**Figures 3D–F**). These results also support that both reduction of leaf ${\text{NO}}_{3}^{-}$ content and higher NUE (**Figure 3D**) resulted from a more efficient ${\text{NO}}_{3}^{-}$ use (NU_T_E; **Figure 3F**), having no effects on ${\text{NO}}_{3}^{-}$ uptake (NU_P_E; **Figure 3E**). From all N forms, organic N represents the main component of TNC in plants, being a crucial element to estimate NUE. We showed that Cl^−^ nutrition increased the organic N content (**Figures 2F**, **3B**), which is in line with the higher NU_T_E values recorded in Cl^−^-treated plants (**Figure 3E**). This points to a greater use of the ${\text{NO}}_{3}^{-}$ accumulated in the leaf, probably as a consequence of the mobilization of the chloroplast ${\text{NO}}_{3}^{-}$ pool to the cytosol, where it can be assimilated (Rosales et al., 2020), supporting the hypothesis that in CL plants, Cl^−^ acquires the osmotic functions of ${\text{NO}}_{3}^{-}$ in the vacuole.

### Chloride stimulates nitrogen assimilation

Previous studies have mostly reported negative responses to Cl^−^ nutrition under salinity conditions (i.e., NaCl), showing a repression of N allocation and assimilation mainly related to the inhibition of the activity and expression pattern of key enzymes such as NR, NiR, GS, GOGAT and GDH in leaves of several crop species (Blom-Zandstra & Lampe, 1983; Barber et al., 1989; Flores et al., 2000; Debouba et al., 2007; Ashraf et al., 2018; Sathee et al., 2021; Zhang et al., 2021). The misconception of using a unique source of Cl^−^ salt (e.g., NaCl or CaCl) alongside the occurrence of ionic or osmotic stress conditions (up to 20 mM), beyond the beneficial macronutrient range (5-6 mM), concealed the impact of Cl^−^ over the cation. Conversely, no NaCl effect on ${\text{NO}}_{3}^{-}$ assimilation was found in leaves of quinoa (Miranda-Apodaca et al., 2020) and *Sophora japonica* (Tian et al., 2022), whereas a positive effect was observed in bean seedlings (Misra and Dwivedi, 1990) and tomato roots (Debouba et al., 2007). Therefore, this is the first report to date that designed a treatment including a mixture of Cl^−^ salts to verify how this ion can be exploited to stimulate N metabolism *via* enzymatic activity in higher plants. To address the involvement of Cl^−^ in this process, we first analysed key enzymes involved in ${\text{NO}}_{3}^{-}$ assimilation pathway. Our results showed that Cl^−^ promoted an increase in the transcript abundance and activity of the NR, supporting the strong decrease of ${\text{NO}}_{3}^{-}$ content in leaves (**Figures 2**, **4**). Likewise, similar Cl^−^-dependent decrease of leaf ${\text{NO}}_{3}^{-}$ content has also been observed in maize (Zhang et al., 2021), tobacco (Rosales et al., 2020), tomato (Neocleous et al., 2021), and indirectly in potato (Abdo et al., 2020). In the chloroplast, NiR activity or expression was not affected by Cl^−^, which is consistent with its constitutive expression reported under biofortification experiments with other ions in lettuce (Blasco et al., 2010; Rios et al., 2010). Enzymes participating in the GS/GOGAT cycle along with cytosolic GDH and AAT also exhibited positive Cl^−^-dependent responses, demonstrating a joint induction at both transcriptional and post-translational levels of the main enzymes involved in the assimilation of ${\text{NH}}_{4}^{+}$ into proteins.

Possibly, the stimulation of the enzymes involved in ${\text{NO}}_{3}^{-}$ assimilation is caused by the higher presence of ${\text{NO}}_{3}^{-}$ in the cytosol induced by the strong Cl^−^ accumulation in the vacuole, which responds to the increase in concentration of the main substrate of this biochemical pathway. Accordingly, our results reveal that Cl^−^ favours protein accumulation in leaves, in detriment of soluble amino acids content (**Figures 5A, B**), whereas chlorophyll a and b remained unchanged (**Figure S2**). This finding could be interpreted as the pool of soluble amino acids is optimized with N assimilation and C fixation by the RuBisCO activity to increase the synthesis of proteins. In fact, the Cl^−^-dependent increase in the gene expression of the RuBisCO, considered as the most abundant protein in higher plants, in addition to other enzymes related with N metabolism and photorespiration (**Figures 4A**, **6B**), supports the greater protein accumulation in 6CL plants.

Interestingly, the results showed a higher content of ${\text{NH}}_{4}^{+}$ induced by the Cl^−^ treatment (**Figure 3C**). This ${\text{NH}}_{4}^{+}$ accumulation is difficult to justify with the higher assimilation of ${\text{NO}}_{3}^{-}$ *via* NR and NiR pathway, since no negative regulation of the GS/GOGAT cycle was found (**Figure 4**) and the content of proteins was higher (**Figure 5A**). These results are in line with those reported by Findenegg et al. (1989), where authors found a higher accumulation of both Cl^−^ and free ${\text{NH}}_{4}^{+}$ in sugarbeet leaves when increasing the Cl^−^ supply from 0.5 to 10 mM in the nutrient solution. ${\text{NH}}_{4}^{+}$ is an intermediate present in several pathways in plants, such as photorespiration, urea and N metabolism, protein or amino acid degradation (Rubio-Wilhelmi et al., 2012; Hildebrandt et al., 2015). Thereby, the analysis of the free amino acids profile can confer new perspectives to elucidate possible metabolic adjustments which remained unknown (Florencio-Ortiz et al., 2018; Batista-Silva et al., 2019; Xu et al., 2022) and, particularly, the rise of ${\text{NH}}_{4}^{+}$ levels in Cl^−^-treated plants (**Figure 3C**). From the amino acids studied, arginine, aspartic acid, glutamic acid, glutamine, Gly, isoleucine and proline were decreased in 6CL plants, which is consistent with the decline of total amino acids content. A further interpretation showed that the Cl^−^-dependent sharp rise of asparagine (**Figure 5C**) might be explained by the dual effect of the N-related enzymes AAT and asparagine synthetase (ASN). In this context, considering the well-known role of Cl^−^ as a cofactor of the ASN enzyme (Rognes, 1980; Marschner and Rengel, 2012; Cakmak et al., 2022), the Cl^−^-dependent up-regulation of the AAT is pursuant to the decrease of aspartic acid levels, one of the ASN substrates. The significant reduction of aspartic acid to synthesize asparagine might directly affect other aspartate-derived amino acids such as methionine, threonine, isoleucine, and lysine (Azevedo et al., 2006), whose contents were not significantly affected by Cl^−^, or slightly reduced as in the case of isoleucine (**Figure 5C**). The conversion of glutamine to glutamic acid and *vice versa* represents the core of the N assimilation pathway, as the α-amino group of glutamic acid is transferred to other amino acids by the action of multispecific aminotransferases (Forde and Lea, 2007; Bender, 2012). In Cl^−^-treated plants, the decrease of the content of both glutamine and glutamic acid for further synthesis of proteins might explain in part the reduction in the efficiency of the GS/GOGAT cycle, even though both enzymes showed increased gene expression and activity levels, and participate in the greater accumulation of ${\text{NH}}_{4}^{+}$ in tobacco leaves. Furthermore, the degradation of arginine generates ornithine and urea, being the latter hydrolyzed by the urease and generating two molecules of ${\text{NH}}_{4}^{+}$ in the cytosol (Winter et al., 2015). Therefore, the decrease in the content of arginine in Cl^−^-treated plants could also participate in the higher ${\text{NH}}_{4}^{+}$ accumulation in leaves. In terms of energetic dynamics, the effort for ${\text{NH}}_{4}^{+}$ reutilization confers fewer requirements of redundant equivalents and intermediates than the ${\text{NO}}_{3}^{-}$ uptake and allocation from roots. The decrease of proline content could be explained since Cl^−^ ameliorates water relations and its role as osmolyte. Thus, the turgor maintenance in Cl^−^ plants might prevent proline biosynthesis (Franco-Navarro et al., 2016; Raven, 2017; Geilfus, 2018). This reinforces the concept that distinguishes Cl^−^ as a beneficial macronutrient versus a promoter of eustress that induces preventive defense responses in plants (Ghosh et al., 2022). Taking all together, this work emphasizes the role of macronutrient Cl^−^ nutrition on the soluble amino acid profile to enhance a more efficient N assimilation in higher plants.

### Chloride application enhances photorespiration yield in higher plants

Far from the traditional paradigm that the photorespiration pathway is a “wasteful” or “futile” cycle, it is now considered one of the most important pathways in plants because it is intimately linked to C-N assimilation, allowing plants to thrive in suboptimal growth environments (Bauwe et al., 2010; Bloom, 2015; Busch et al., 2018). Photorespiration participates in the assimilation of N, considering that the major ${\text{NH}}_{4}^{+}$ assimilated by GS/GOGAT comes from photorespiration rather than from ${\text{NO}}_{3}^{-}$ assimilation (Keys, 2006; Masclaux-Daubresse et al., 2010; Umnajkitikorn et al., 2020). Additionally, photorespiration provides intermediates and reduced equivalents for the Krebs cycle and it is a sink for ROS elimination and energy dissipation to photoinhibition avoidance (Wingler et al., 2000; Maurino & Peterhansel, 2010; Wada et al., 2021). Then, the depletion of the photorespiration pathway could strongly condition plant survival, being suggested as a target for molecular genetic engineering in agriculture to improve crop yield (Betti et al., 2016). Consistently, Busch et al. (2018) demonstrated that photosynthetic CO_2_ uptake increases when assimilating N *via* the photorespiratory pathway, by fixing C as amino acids in addition to carbohydrates. Thus, photorespiration favors the metabolism of N, C and sulfur, generating Ser (*i.e.,* a precursor of cysteine) from Gly decarboxylation (Shi & Bloom, 2021). Surprisingly, the results of the amino acid profile revealed a strong reduction in the Gly/Ser ratio in Cl^−^-treated plants (**Figure 6A**), indicating a stimulation of the formation of ${\text{NH}}_{4}^{+}$ , CO_2_ and NADPH in the mitochondria. Consistently, the Cl^−^-dependent increase in the expression patterns of key photorespiration-related enzymes supported the stimulation of the photorespiratory pathway under macronutrient Cl^−^ conditions (**Figure 6B**). The physical association between chloroplasts, peroxisomes, and mitochondria is important in the regulation of the photorespiratory process (Rivero et al., 2009; Shai et al., 2018). TEM micrographs made in both 0CL and 6CL plants (**Figure 6C**) clearly showed that Cl^−^ favours the interaction between peroxisomes, mitochondria and chloroplasts (**Figure 6**). Therefore, these results strongly support the higher activity of the photorespiration pathway in Cl^−^-treated plants.

**Figure 7:**
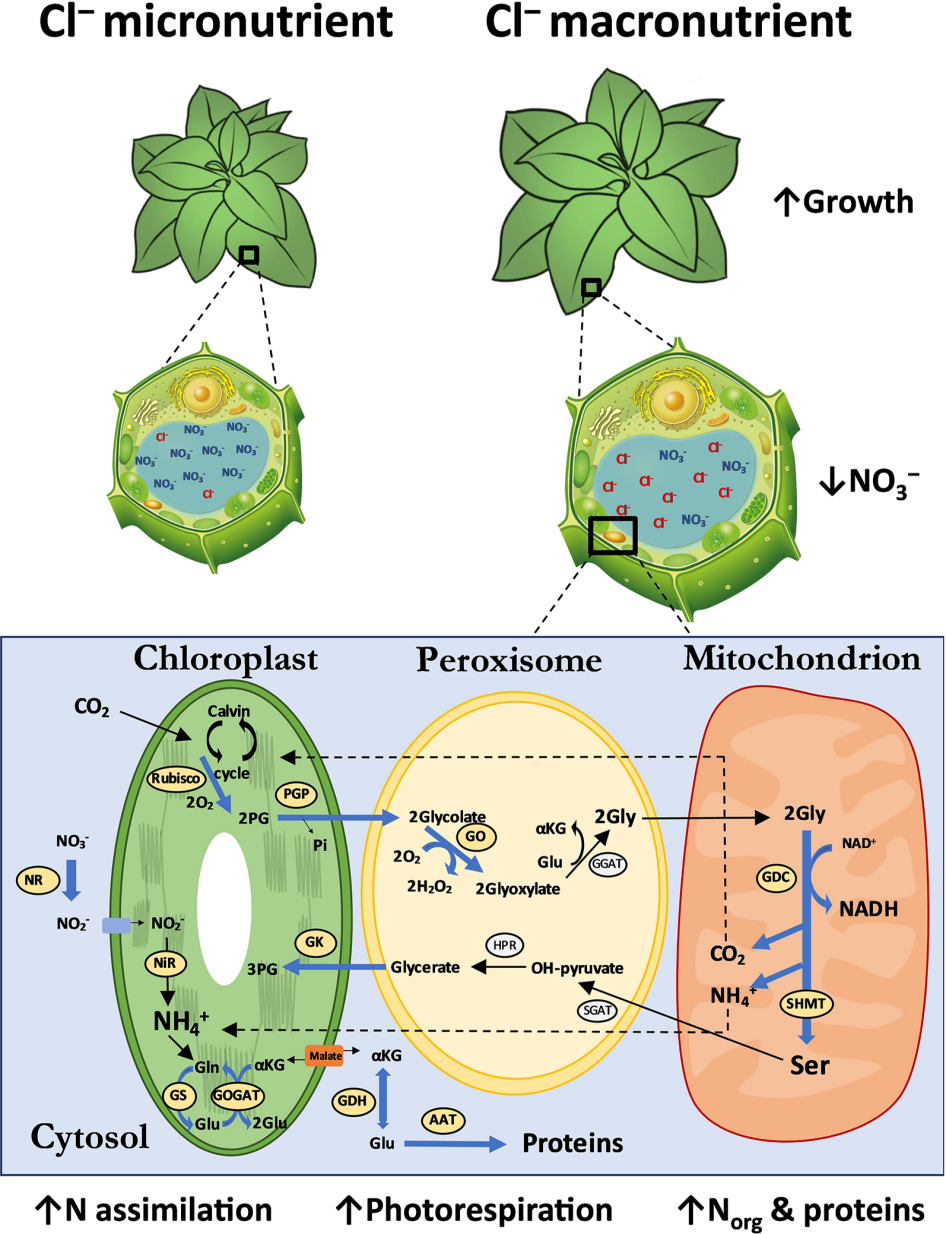
Integrative model of anatomical, cellular and metabolic responses to Cl^−^ nutrition at macronutrient levels. The 0 and 6 mM Cl^−^ treatments containing similar cationic balance are represented as Cl^−^ micronutrient (0.075 mM Cl^−^) and Cl^−^ macronutrient (6.075 mM Cl^−^). The leaf anatomical and cellular parameters (Franco-Navarro et al., 2019), and N metabolism analyzed are schematically represented. The blue arrows represent the up-regulatory enzymatic responses observed. The font size of the analyzed molecules represents their abundance under Cl^−^ treatment. AAT, aspartate aminotransferase; GDC, glycine decarboxylase; GDH, glutamate dehydrogenase; GGAT, glutamate:glyoxylate aminotransferase; GK, glycine kinase; Gln, glutamine; Glu, glutamic acid; Gly, glycine; GO, glycolate oxydase; GOGAT, glutamate synthase; GS, glutamine synthetase; HPR, hydroxypyruvate reductase; NAD, nicotinamide adenine dinucleotide; NiR, nitrite reductase; NR, nitrate reductase; PG, 3-phosphoglycerate; PGP, phosphoglycolate phosphatase; RuBisCO, ribulose-1,5-biphosphate carboxylase/oxygenase; Ser, serine; SGAT, serine:glyoxilate aminotransferase; SHMT, serine hydroxymethyl transferase; αKG, α-Ketoglutaric acid.

The co-ordination of C and N assimilation has been studied extensively over many years because it is a key determinant of plant productivity. The integration of primary N assimilation with the re-assimilation of ${\text{NH}}_{4}^{+}$ produced by photorespiration, involves a complex network of molecular, physiological and metabolic controls that modify the activities of components of both C and N metabolism (Ferrario-Mery et al., 2002), being the phosphoenolpyruvate carboxylase (PEPC) one of the enzymes involved in these processes. In the C_3_ metabolism, PEPC is a cytosolic enzyme that catalyses the irreversible β-carboxylation of phosphoenolpyruvate in the presence of HCO^−^ and Mg^2+^ to yield phosphate and oxalacetate (Hatch & Kagawa, 1973), and participates in non-photosynthetic processes including the anaplerotic flux replenishing intermediates to the Krebs cycle (Miyao and Fukayama, 2003; O’Leary et al., 2011). In this work, the activity of cytosolic PEPC in Cl^−^-treated plants significantly increased in tobacco leaves (**Figure S3**), in line with the higher N assimilation and photorespiration previously observed. Even though PEPC is not a key enzyme in CO_2_ assimilation as it is in C_4_ metabolism, the C/N ratios must be compensated given the stimulation of N metabolism. Accordingly, the PEPC activity also correlated with the higher relative transcript abundance of the RuBisCO in Cl^−^-treated plants (**Figure 6**). Previous appreciations by Franco-Navarro et al. (2019) underlined that larger mesophyll cells of Cl^−^-treated tobacco plants lead to a reduction of stomatal conductance (*g*_s_) without impairing photosynthesis rates (*A*_N_), and improving water use efficiency (WUE). Nonetheless, the low *g*_s_ rates were counteracted by the higher mesophyll diffusion conductance to CO_2_ (*g*_m_), avoiding a negative effect on photosynthetic performance, due to a higher surface of chloroplasts exposed to the intercellular airspace of mesophyll cells. Consistently, Busch et al. (2018) demonstrated that photosynthetic CO_2_ uptake increases when assimilating N *via* the photorespiratory pathway, by fixing C as amino acids in addition to carbohydrates. Therefore, these results support that the stronger Cl^−^-dependent assimilation of N is coordinated with a higher assimilation of C to improve growth in tobacco plants. Taken together, and after having ruled out the occurrence of Cl^−^/ ${\text{NO}}_{3}^{-}$ antagonism, we show in this work that the leaf ${\text{NO}}_{3}^{-}$ decrease is a consequence of the specific stimulation by Cl^−^ of biochemical pathways involved on ${\text{NO}}_{3}^{-}$ assimilation and photorespiration, as shown in the model represented in the **Figure 7**. To better understand the specific roles of Cl^−^ over the key genes of photorespiration pathway, deeper research must be done. As a future perspective, the deletion/overexpression of RuBisCO subunits, SHMT or GO genes in transformed tobacco lines could clearly demonstrate the induction of macronutrient Cl^–^ and therefore reinforce the current evidence observed in our research. The complex interaction of this process with chloroplast, peroxisomes, mitochondria, and cytosol compartments led us to believe that macronutrient Cl^–^ could acquire other specific roles in higher plants.

## Conclusion

Finally, we conclude in this work that Cl^−^ nutrition supplied at beneficial macronutrient levels improves NUE *via* the concurrent induction of both ${\text{NO}}_{3}^{-}$ assimilation and photorespiration pathways. The changes in N forms in shoot are well justified by the effect of Cl^−^ on the synthesis and activity of enzymes involved in these N-metabolism pathways. The increment of ${\text{NH}}_{4}^{+}$ and the significant reduction in the Gly/Ser ratios indicate the involvement of photorespiration in the metabolic responses to Cl^−^ nutrition. And the higher organic N content in leaf tissue combined with higher content of soluble proteins and lower content in free amino acids in Cl^−^-treated plants indicate that Cl^−^ promotes the synchronized assimilation of N and C, therefore producing higher biomass in plants. Thus, we propose that the optimal Cl^−^ implementation in crop fields could be useful to improve biomass production even when N availability is scarce. Therefore, Cl^−^ must be considered a good candidate to reduce both N application in farmlands and deleterious ${\text{NO}}_{3}^{-}$ content in vegetables.

## Data availability statement

The raw data supporting the conclusions of this article will be made available by the authors, without undue reservation.

## Author contributions

PP-T, RA, ML, JDF-N, and FD-G performed the experiments, participated in the conception of experiments and research plans, analyzed and plotted the data; PP-T participated in the writing of the article; JC-F participated in the conception of research plans, co-funded to finance the project, supervised the experiments, and participated in the writing of the article; and MR supervised and participated in the performance of the experiments, conceived research plans, co-funded the project and wrote the article. All authors contributed to the article and approved the submitted version.
