# Supplementary material for: Nitrogen assimilation and photorespiration become more efficient under chloride nutrition as a beneficial macronutrient
Source: Front Plant Sci. 2023 Jan 10;13:1058774. doi: 10.3389/fpls.2022.1058774 (PMC9871469; doi:10.3389/fpls.2022.1058774)
Supplement: Supplementary file 1 [file DataSheet_1.pdf]

Table S1. Primers and genes identification of N metabolism-related enzymes.

Gene	Forward	Reverse
NtNR<br>Nitab4.5_0023821g0010.1	CCAAGAGAGAAAATCCCATGC	CTTGACAACCAACTCGAAGTAC
NtNiR<br>Nitab4.5_0005249g0010.1	CAATATGGGCGGTTCATGATG	GCTAGTCCCTTTAGTATCTCCG
NtGS<br>Nitab4.5_0003070g0010.1	CAAGTAGGTCCTAGTGTGGG	TCAAATCCTCCCTCTTCTCTC
NtGOGAT<br>Nitab4.5_0002492g0040.1	CTTCCTACCTACCTCTGAAAG	GAAACACTTGCTCAATGACAGG
NtGDH<br>Nitab4.5_0002359g0020.1	GGCTAAGTTCATCATAGAAGCAG	GGTTATGGAAGGCTTTTGTCATG
NtAAT<br>Nitab4.5_0007126g0030.1	CTCGGCGTCACTATTGCTTAC	CGAGACCTGTCATTTACTAGTAG
NtASN<br>Nitab4.5_0008156g0030.1	GAGCAAAGGAGGATTGAAAAGTG	GTAATGGGTGTGTTATGAGGG
NtL25<br>Nitab4.5_0002995g0050.1	GATTGAGGACAACAACACCCTTG	TCCAAAGCATCATAGTCAGGAG
NtGAPDH<br>Nitab4.5_0005327g0090.1	CAAATTGCCTTGCTCCCTTGGC	CAGCCTTGGCAGCTCCAG

Experimental procedure: List of primers for N metabolism related enzymes: nitrate reductase (NR), nitrite reductase (NiR), glutamine synthetase (GS), glutamate synthase (GOGAT), glutamate dehydrogenase (GDH), and aspartate aminotransferase (AAT). The accesion number from each *N. tabacum* candidate genes were selected using the Solanaceae Genomics Network database (https://solgenomics.net/). Further analysis related to the design of qPCR primers assays were obtained following Primer3 and Oligocalc software procedures.

Table S2. Primers and genes identification of photorespiration-related enzymes.

Gene	Forward	Reverse
NtRuBP Nitab4.5_0000922g0060.1	AATACGATGCTGCTCTTCTTGATGC	GATAGGACAACTCTCAAGGCTGC
NtPGP Nitab4.5_0001003g0100.1	CTTCATGATGGACTACTTGGC	CTTGTTGGCGTAGAAATCTGG
NtGO Nitab4.5_0001924g0080.1	CCAACTCGATTATGTCCCTTC	CAGCAGCCAATGAGAAAACTAC
NtGDC Nitab4.5_0003627g0010.1	GGTGAAATCTTGGACTATGGG	GATTCTTCCGGGCATCATTCTC
NtSHMT1 Nitab4.5_0000816g0150.1	GGAGAGATACTGATAGCACGTCG	CCCTTTGTACTGTCTCTGCTTCTC
NtSHMT2 Nitab4.5_0003358g0060.1	CTGTTATCGCGTTTCTTGGTTTG	CATTCGTGGTACTCATTTTGGAC
NtSHMT3 Nitab4.5_0000036g0440.1	GAGGTGTTGTACGACTATGAAG	ACCAGAAACAAGCTCATAACCC
NtGK Nitab4.5_0005784g0040.1	CCAGTCTTCTTGTGGTGTGAAC	CGGGTTGTCCTCTCTTAGTTTG

Experimental procedure: List of primers for photorespiration-related enzymes; ribulose-1,5-biphosphate (RuBP), phosphoglycolate phosphatase (PGP), glycolate oxydase (GO), glycine decarboxylase (GDC), serine hydroxymethyl transferase (SHMT), glycine kinase (GK). The accesion number from each *N. tabacum* candidate genes were selected using the Solanaceae Genomics Network database (https://solgenomics.net/). Further analysis related to the design of qPCR primers assays were obtained following Primer3 and Oligocalc software procedures.

Table S3. Relation of nutritional treatments.

Nutrient content in treatments (mM)

Nutrients	0CL	2CL	6CL
	0:8 (SP: CL)	2:6 (SP: CL)	6:2 (SP: CL)
Na^+	0.1	0.1	0.1
K^+	9.28	8.96	8.32
Ca^{2+}	2.6	2.45	2.15
Mg^{2+}	2.6	2.45	2.15
Cl^-	0.075	2.075	6.075
NO_3^-	8	8	8
SO_4^{2-}	3.2	2.4	0.8
PO_4^{3-}	4.8	3.6	1.2
S+P	8	6	2

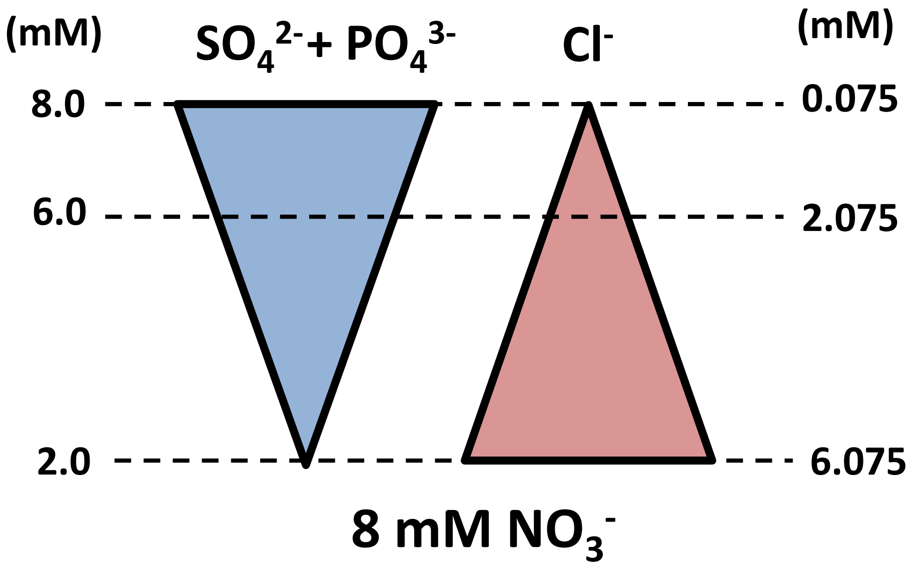

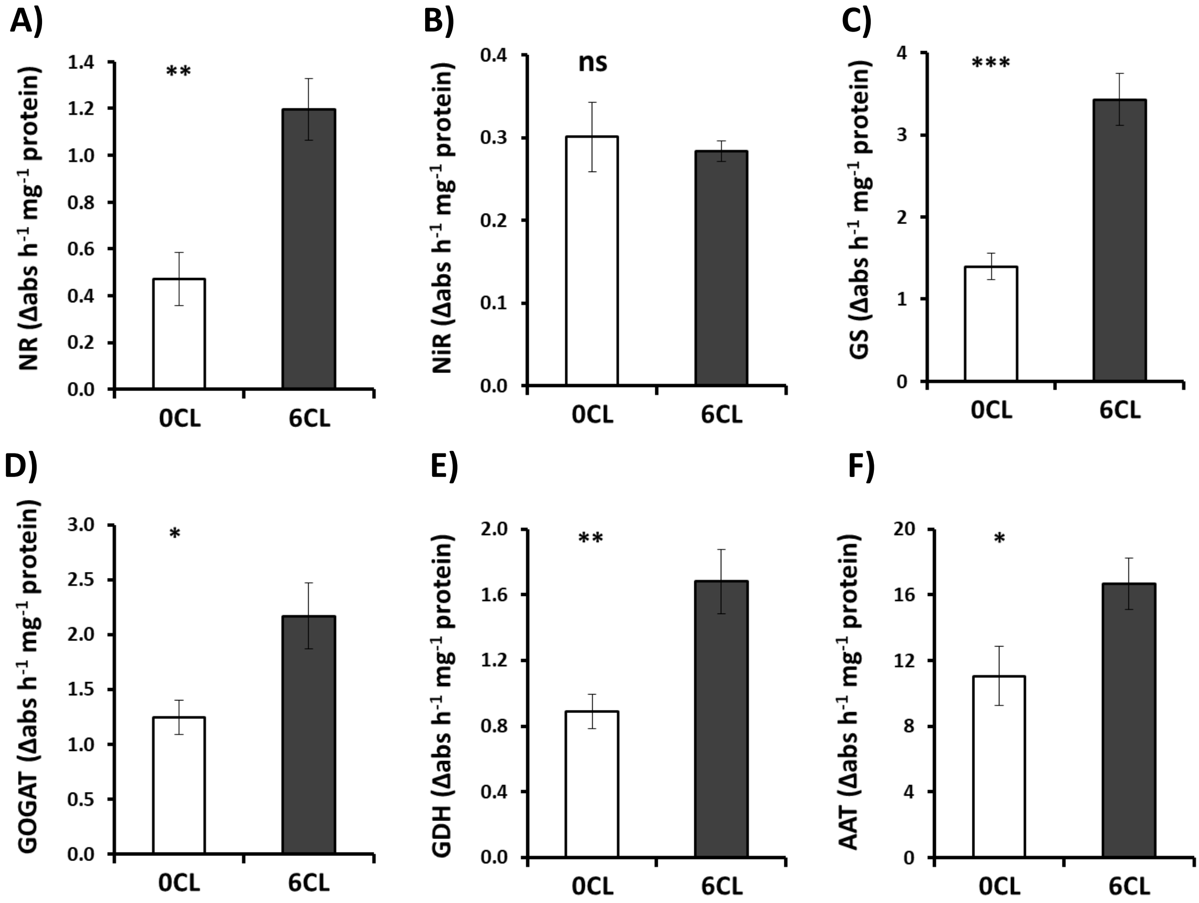

Figure S1. Effect of Cl^- nutrition on N metabolism enzymes. Tobacco plants were treated with 0 and 6 mM of Cl^- salts (CL), and supplemented with a mix of sulphate+phosphate salts to maintain a similar cationic balance. **(A)** Nitrate reductase (NR); **(B)** nitrite reductase (NiR); **(C)** glutamine synthetase (GS); **(D)** glutamate synthase (GOGAT); **(E)** glutamate dehydrogenase (GDH); and **(F)** aspartate aminotransferase (AAT). Mean values ±SE, n=4. Statistics was calculated through ANOVA. Significance: ns, not significant; *P≤0.05, **P≤0.01, and ***P≤0.001.

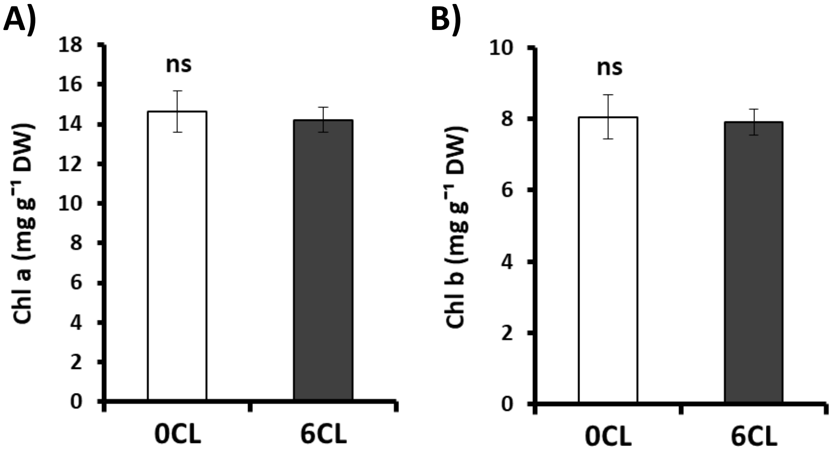

Figure S2. Effect of Cl^- nutrition on chlorophylls and carotenoids. Tobacco plants were treated with 0 and 6 mM of Cl^- salts (CL), and supplemented with a mix of sulphate+phosphate salts to maintain a similar cationic balance. **(A-C)** Content of chlorophyll a (chl a) and b (chl b) was expressed by mg per gr of dry weight (DW).Mean values ±SE, *n*=4-6. Statistics was calculated through ANOVA. Significance: ns, not significant, and *$P \leq 0.05$.

<u>Experimental procedure.</u> Determination of pigments: Frozen tissues were used for the analysis of photosynthetic pigments following the method of Litchenthaler (1987; J. Plant Physiol. 131:101). Briefly, samples were grinded with 80% acetone in chilled mortar and centrifuged at 30,000 *g*. The supernatant obtained was measured spectro-photometrically. Results were expressed in mg of pigment per mg of dry weight (DW).

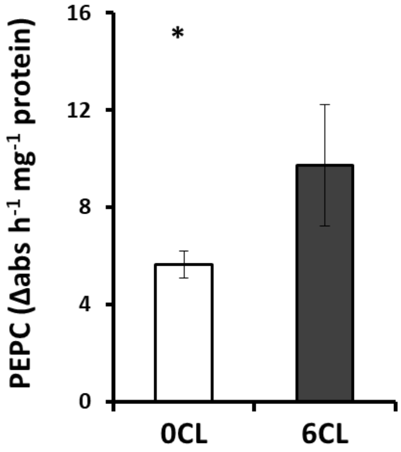

Figure S3. Effect of Cl^{-} nutrition on phosphoenolpyruvate carboxylase (PEPC). Tobacco plants were treated with 0 and 6 mM of Cl^{-} salts (CL), and supplemented with a mix of sulphate+phosphate salts to maintain a similar cationic balance. **(A)** phosphoenolpyruvate carboxylase (PEPC). Mean values ±SE, n=6. Statistics was calculated through ANOVA. Levels of significance: *$P \leq 0.05$.

Experimental procedure. A quantity of 0.2 g of frozen leaf tissue was grounded in a chilled mortar with 1 mL of extract buffer which contained 0.1 M Tris-HCl (pH 7.5), 20% (v/v) glycerol, 1 mM EDTA, 10 mM $MgCl_2$, 14 mM mercaptoethanol. Homogenate was centrifuged for 2 min at 15.000 rpm and supernatant was used to determine the activity of the PEPC (EC 4.1.1.31) enzyme. PEPC activity was measured spectrophotometrically at pH 8.0 using the NAD-malate dehydrogenase-coupled assay at 2.5 mM phosphoenolpyruvate (Echevarria *et al.*, 1994; Arch. Biochem. Biophys. 315: 425). Protein assay was done according to the colorimetric method Bradford (1976; Anal. Biochem. 72:248) using bovine serum albumin (BSA) as standard.

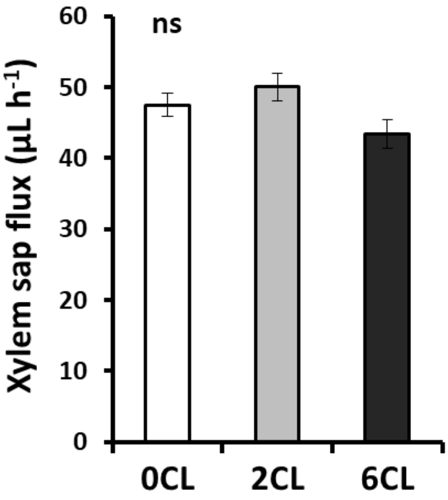

Figure S4. Effect of Cl⁻ nutrition on xylem sap exudates. Tobacco plants were treated with 0, 2 and 6 mM of Cl⁻ salts (CL), and supplemented with a mix of sulphate+phosphate salts to maintain a similar cationic balance. Xylem sap flux per hour is represented. Mean values ±SE, *n*=6. Statistics was calculated through ANOVA. Significance: ns, not significant; *P≤0.05.

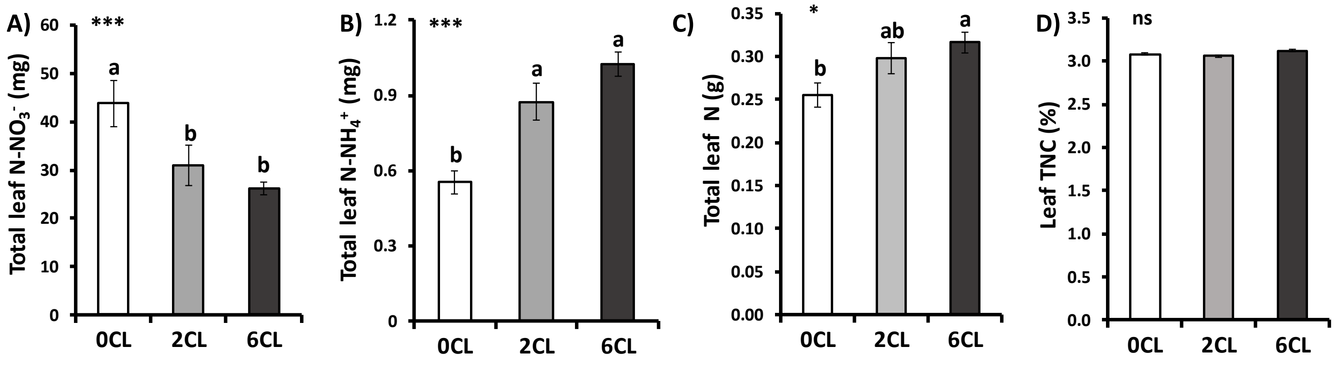

Figure S5. Effect of Cl− nutrition on nitrogen assimilation in leaves. Tobacco plants were treated with 0, 2 and 6 mM of Cl− salts (CL), and supplemented with a mix of sulphate+phosphate salts to maintain a similar cationic balance. A) mg of $N\text{-}NO_3^-$ content per total leaf dry biomass, B) mg of $N\text{-}NH_4^+$ content per total leaf dry biomass, C) g of total nitrogen per total leaf dry biomass and D) Percentage of Total Nitrogen Content (TNC) in 100 gr of dried leaf biomass. Mean values ±SE, n=6. Statistics was calculated through ANOVA. Levels of significance: *P≤0.05, ***P*≤0.01, and ****P*≤0.001.
